# What drives health care spending in Switzerland? Findings from a decomposition by disease, health service, sex, and age

**DOI:** 10.1186/s12913-023-10124-3

**Published:** 2023-10-25

**Authors:** Michael Stucki, Xavier Schärer, Maria Trottmann, Stefan Scholz-Odermatt, Simon Wieser

**Affiliations:** 1 ZHAW Zurich University of Applied Sciences, Winterthur Institute of Health Economics, Gertrudstrasse 8, Winterthur, 8401 Switzerland; 2 Department of Health Sciences and Medicine, University of Lucerne, Lucerne, Switzerland; 3 SWICA Health Care Organisation CH, Winterthur, Switzerland; 4 Suva Swiss National Accident Insurance Fund, Lucerne, Switzerland

**Keywords:** Health care spending, Spending growth, Cost-of-illness, Switzerland, Spending decomposition, I10

## Abstract

**Background:**

High and increasing spending dominates the public discussion on healthcare in Switzerland. However, the drivers of the spending increase are poorly understood. This study decomposes health care spending by diseases and other perspectives and estimates the contribution of single cost drivers to overall healthcare spending growth in Switzerland between 2012 and 2017.

**Methods:**

We decompose total healthcare spending according to National Health Accounts by 48 major diseases, injuries, and other conditions, 20 health services, 21 age groups, and sex of patients. This decomposition is based on micro-data from a multitude of data sources such as the hospital inpatient registry, health and accident insurance claims data, and population surveys. We identify the contribution of four main drivers of spending: population growth, change in population structure (age/sex distribution), changes in disease prevalence, and changes in spending per prevalent patient.

**Results:**

*Mental disorders* were the most expensive major disease group in both 2012 and 2017, followed by *musculoskeletal disorders* and *neurological disorders*. Total health care spending increased by 19.7% between 2012 and 2017. An increase in spending per prevalent patient was the most important spending driver (43.5% of total increase), followed by changes in population size (29.8%), in population structure (14.5%), and in disease prevalence (12.2%).

**Conclusions:**

A large part of the recent health care spending growth in Switzerland was associated with increases in spending per patient. This may indicate an increase in the treatment intensity. Future research should show if the spending increases were cost-effective.

**Supplementary Information:**

The online version contains supplementary material available at 10.1186/s12913-023-10124-3.

## Introduction

High income countries spend a substantial and increasing share of their income on health care. Health care spending is particularly high in Switzerland, with a share of 11.8% in gross domestic product (GDP) in 2020, and per capita spending at 7,179 purchasing power parity adjusted US dollars, second only to the United States (US) [1]. Possible drivers of spending growth include ageing populations [2, 3], increasing disease prevalence, increasing incomes [4, 5], increasing prices [6, 7], increasing intensity of treatment, new treatments and drugs [5], and increasing overuse [8]. However, the contribution of each of these factors to the overall spending growth remains unknown.

The existing literature has mostly aimed to identify the drivers of health care spending in highly aggregated spending data. Only few studies have taken the different approach of first decomposing health spending by different perspectives, in particular by diseases, and only then assessing the contribution of different drivers to overall spending growth [9–11]. For simplicity, in this paper we use the term diseases for all health conditions, including illness, injuries, impairment, and other reasons for the use of health services such as well care (e.g., check-ups).

A decomposition of health care spending has several advantages: *First*, it allows a more detailed assessment of spending drivers, as some important drivers are difficult to operationalize at the aggregate level (e.g., the prevalence of different diseases) and as the effect of some drivers can be lost due to composition effects (e.g., contrasting price trends in health services). *Second*, it permits a detailed monitoring of spending by disease and other perspectives, such as by health services, payers, and age groups. The combined information of these perspectives may reveal valuable information, such as changes in the way a disease is treated. *Third*, the results of the decomposition may serve as the basis for other research such as the comparison of spending with health outcomes at the disease level. *Finally*, a better and more detailed understanding of the spending drivers may contribute to the definition of appropriate measures for cost containment.

Recent research has demonstrated the benefits of decomposing total health care spending by diseases and other factors [10–18]. There are only few studies which decomposed spending in Switzerland by disease [13–15]. A main limiting factor is the lack of diagnostic coding in outpatient care and long-term inpatient care.

Switzerland has a market-oriented health care system with a multitude of service providers and financing regimens. Mandatory health insurance (MHI) provides a generous benefits package provided by private non-profit insurers and is supplemented by other social insurance schemes, such as accident and disability insurance. The federal government oversees legislation, but most health care provision is organized at the sub-federal level in the 26 cantons. This decentralized structure is reflected in a lack of comprehensive individual-level data on health service use and spending, with the only exception of the national hospital inpatient registry (HospReg) [19]. However, the Swiss health care system has an important strength facilitating the decomposition of spending by disease: A high uniformity and transparency of the prices and coding of health services and products is assured by several nationally uniform tariffs as well as national tariff lists (e.g., for drugs) released by the federal government.

This paper has two goals. *First*, to decompose total health care spending for 20 distinct services and drugs according to the Swiss National Health Accounts (NHA) by an exhaustive set of 48 mutually exclusive diseases or disease groups, sex, and age groups in 2012 and 2017. *Second*, to decompose the change in health care spending over time into four fundamental factors: population growth, change in population structure (age/sex distribution), change in disease prevalence, and change in the average spending per prevalent patient.

We contribute to the existing literature in multiple ways. *First*, we improve on previous studies decomposing Swiss health care spending by diseases [14, 15] by substantially increasing the granularity of the decomposition. *Second*, we apply a comprehensive approach by using an exhaustive and mutually exclusive list of medical conditions, including diseases, injuries, and well care. We thereby avoid double-counting, which is a well-known drawback of single cost-of-illness studies [20]. *Third*, we use the highly granular spending decomposition in 2012 and 2017 to identify the contribution of four factors to the increase in health care spending over this period.

## Data and methods

**Fig. 1:**
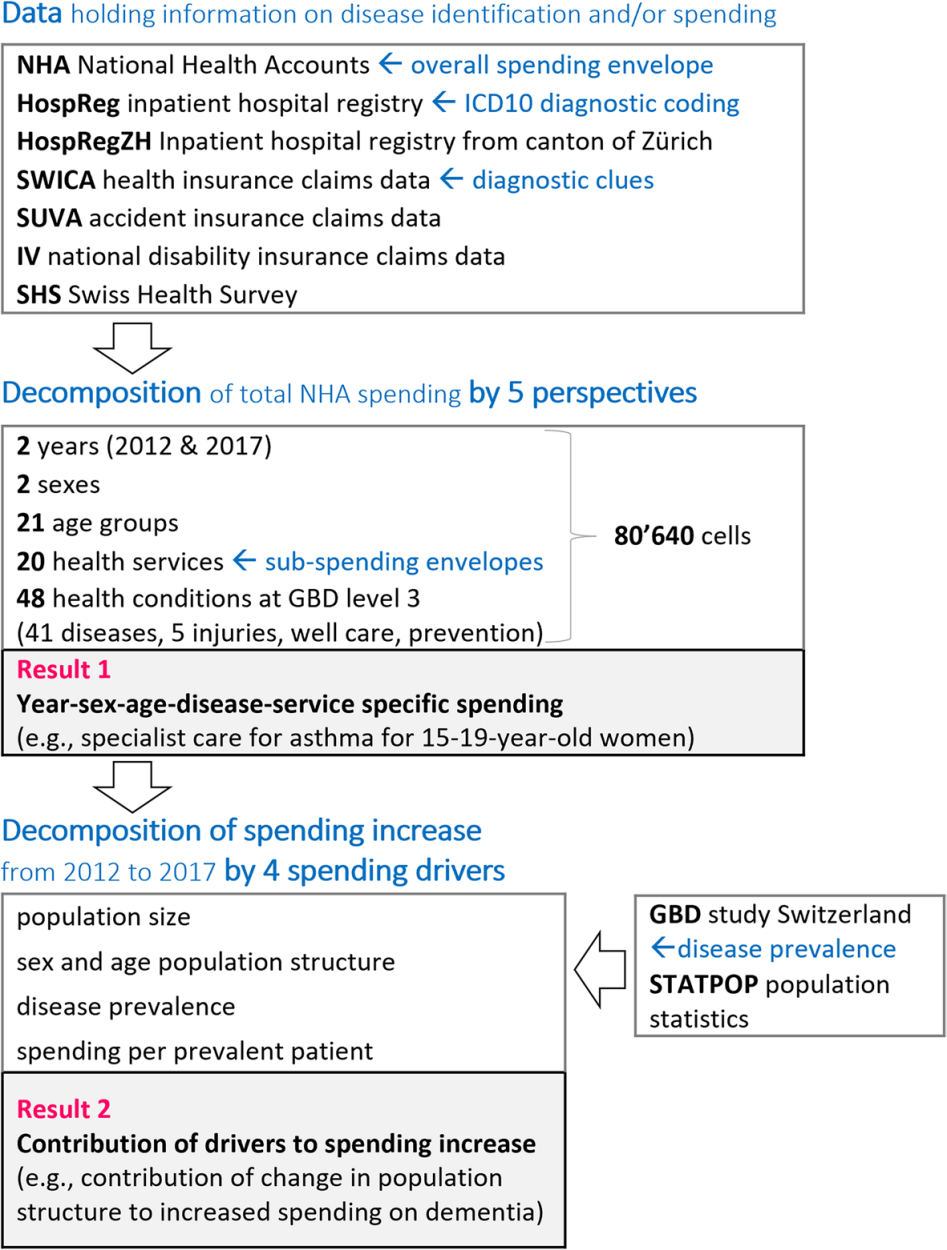
Overview of data and methods. GBD: Global Burden of Disease Study

Figure 1 gives an overview of data and methods used in the two steps of the study. We first decomposed total NHA spending by five perspectives using various sources of micro-data. The results of this decomposition were then used to assess the contribution of four spending drivers on the overall and disease-specific spending increase from 2012 to 2017. Parts of this approach have been previously described [13, 14].

### Data

We used a variety of data sources to identify diseases and estimate spending by diseases and other perspectives. Table 1 provides an overview of the data used. Further details are reported in Estimation of disease-specific spending by type of health service section and in the supplementary material (Online resource 1).

**Table 1:** Overview of data sources used in decomposition of spending

**Data source and provider**	**Description of data source**	**Information used for study**	**Comments**
National Health Accounts NHA (FSO) [21]	Total yearly health care spending by four perspectives (health services, health service providers, financing regimes, payers)	Total spending by service, service provider, and payer	The list of services was modified to obtain 20 distinct and comprehensive services. Some service categories were aggregated, while others were created by combining types of services and providers.
Inpatient hospital registry HospReg (FSO) [19]	Complete registry of all inpatient cases in acute care hospitals and psychiatric and rehabilitation clinics. Includes ICD-10 diagnoses and procedures according to Swiss classification of surgical interventions CHOP	Sex, age, ICD-10 diagnoses, transfer to nursing home, case-level information such as length of stay or DRG cost weight for assignment of spending for inpatient services covered by MHI and other social insurance schemes	HospReg uses the ICD-10-GM (German Modification) which, for the purposes of this study, is largely comparable to the ICD-10 classification of the World Health Organization.
Inpatient hospital registry from canton of Zurich HospRegZH (Zurich Cantonal Department of Health)	Complete registry of all inpatient treated cases in acute care hospitals in the canton of Zurich. In addition to variables in HospReg, it also includes case-level costs (costs of service provision).	Sex, age, ICD-10 diagnoses, to adjust the spending for each case for the presence of comorbidities.	Only used as auxiliary input to the comorbidity adjustment in inpatient acute somatic care.
Swiss Health Survey SHS (FSO) [22]	Large representative population survey on health status, health behaviour, health care utilization, conducted every five years since 1992	Prevalence of *osteoporosis* by sex and age; number of dentist visits by sex and age	
SWICA Health insurance claims data (data extraction for this study)	Full claims data for treatments covered by MHI, full claims data for treatments covered by accident insurance for non-work force individuals without compulsory accident insurance under Accident Insurance Act	Prevalence of ‘other’ (residual) disease categories and well care; spending for all outpatient services covered by MHI; spending for inpatient long-term care covered by MHI, injury spending covered by MHI by age, sex, and service provider, prevalence of *other injuries* by sex and age	SWICA is a large representative MHI insurer with a market share of 8.1% in 2017.
Suva Accident insurance claims data (data extraction for this study)	Full claims data for treatments covered by accident insurance for work force individuals	Spending for all outpatient and inpatient services covered by accident insurance (mainly injuries) Number of cases by year, injury category, age, and sex	Suva is a large public accident insurer covering around 50% of the national work force, mainly in industrial production, construction, and transport.
IV mandatory national disability insurance claims data (Federal Social Insurance Office) (data extraction for this study)	Aggregated health care data by sex, age, type of health service, category of disability	Spending for all outpatient and inpatient services covered by disability insurance by sex and age	IV covers all health care spending for the treatment of congenital birth defects until the age of 20 years.
Global Burden of Disease study [23]	Global study on epidemiology of a wide range of diseases, injuries, and risk factors. Includes detailed estimates for Switzerland.	Prevalence rates by year and disease by sex and age	Study measures overall prevalence, not only treated prevalence.
STATPOP population statistics (FSO) [24]	Yearly statistics on the size and structure of the Swiss population	Population size by year, sex, and age	

*FSO* Federal Statistical Office, *MHI* Mandatory health insurance

The overall envelope of our spending decomposition was given by the NHA provided by the Swiss Federal Statistical Office (FSO) according to OECD standards [21]. The Swiss NHA assess total yearly spending by the four perspectives of health services, health service providers, financing regimes, and payers. We calibrated our estimates of diseases-specific spending by health service to the total of each health service according to NHA. This ensured that the spending proportions attributed to each disease were the same in total and in the micro-data used for spending attribution. This calibration was particularly important when the micro-data did not cover the full population, as in the case of claims data from a single insurer.

### Overview: methods of spending decomposition

We defined a decomposition framework of all five perspectives (year, sex, age groups, diseases, health services):

- The decomposition was carried out for the years 2012 and 2017, as all data was available for both years, and in particular the SHS performed only every 5 years.
- We distinguished 21 age groups: 0 years, 1–4 years, 5-year age groups from 5 to 94 years, 95+ years.
- Health services were defined based on the NHA classification [21] and distinguished by five broad service categories and 20 more specific health services (Table 2). 16 of these corresponded to NHA service types. In addition, we split the NHA service physician outpatient by general practitioners (GP) / specialists and the NHA service rehabilitation outpatient by physiotherapists / occupational therapists. Total spending for each service and year according to NHA is provided in the supplementary material (Online resource 1).
- Diseases were classified according to the exhaustive and mutually exclusive Global Burden of Disease (GBD) classification [25]. This classification has several advantages: *First*, it allows for a mapping of ICD-10 codes to the disease categories. *Second*, it has been used in similar research [11, 15], which enables a comparison of our results to previous studies. *Third*, the GBD study includes sex and age specific estimates of disease prevalence rates which we used for the decomposition of the spending changes over time.

**Table 2:** Classification of health services based on National Health Accounts

**Broad health service categories**	**More specific health services**	**Comment**
Outpatient	Physician services (general practitioners)	
	Physician services (specialists)	
	Hospital outpatient	
	Drugs outpatient	prescription and over-the-counter
	Psychotherapy and psychiatry	
	Physiotherapy	
	Occupational therapy	
	Dental care	
	Medical devices and products	
	Long-term home-care	
	Other outpatient care	
Other outpatient care	Laboratory tests	
	Radiology	
	Ambulance and rescue	
Inpatient care	Acute somatic care	
	Rehabilitation	
	Psychiatry	
	Long-term care in nursing homes	we distinguished between nursing homes and institutions for people with addictions
Administration	Administration	includes health and accident insurers and public health care administration
Prevention	Prevention	includes prevention and health promotion by public and private agencies, but not by health service providers

The GBD study classifies diseases hierarchically at four different levels. GBD level 1 makes a broad distinction between *communicable diseases* (including *nutritional deficiencies* and *maternal/neonatal disorders*), *non-communicable diseases* (NCDs), and *injuries*. We added *well care* at this level, as health services are also used for other reasons than diseases, such as pregnancy without complications. GBD level 2 distinguishes between major diseases within level 1, such as *cardiovascular diseases* or *neoplasms* within *NCDs*. GBD levels 3 and 4 distinguish single diseases within major diseases, such as *ischemic heart disease* or *stroke* within *cardiovascular diseases*.

We used a simplified GBD classification as, due to a lack of diagnostic coding, it was impossible to identify all the 369 diseases of the GBD study. Our simplified exhaustive and mutually exclusive disease classification consisted of 16 major disease categories at GBD level 2, and 41 diseases, five injuries and *well care* at GBD level 3. *Prevention* was defined as an additional reason for health care spending. Our classification of injuries differed from the GBD classification, as Swiss accident insurance data distinguish between *road injuries* and *other injuries*, each split by their context into *occupational* or *non-occupational* injuries. A fifth injury type comprises *residual injuries* that we could not assign to one of the other four types.

Combining the 2 years, 2 sexes, 21 age groups, 20 health services and 48 conditions resulted in a multi-dimensional grid of 80,640 cells to be filled with estimates. Some cells were left empty because the combination was unfeasible (e.g., *prostate cancer* in women) or because the decomposition was not possible for one or more perspectives (e.g., *prevention* by diseases and sex/age). Whenever useful, we included the payer’s spending share for each service type according to NHA in the decomposition. We evaluated the number of observations used in the estimation of spending in cells for which we only had a sample of the population (e.g., claims data from MHI).

We applied a bottom-up approach to assign spending to diseases, complemented with top-down assignments when no micro-data was available. Bottom-up assignment was based on patient-level micro-data, such as health and accident insurance claims data. Whenever the sum of spending assigned based on micro-data was different from the total for that service type given by the NHA, we re-scaled the spending to meet the total. Top-down assignment was based on disease-specific spending information contained in NHA. This was the case for outpatient dental care, which was assigned to *oral disorders*, and expenditures by the mandatory disability insurance covering spending for the treatment of *congenital birth defects* until the age of 20 years. Spending for administration in NHA was assigned in proportion to the disease-specific spending resulting from the bottom-up assignment.

### Estimation of disease-specific spending by type of health service

The micro-data-based assignment of spending to diseases required two steps for each type of health service: *First*, the identification of the relevant diseases and, *second*, the allocation of the ‘right’ amount of spending to each disease. Online resource 1 provides the details on the methodology for each health service.

#### Outpatient care and diagnostics, and drugs

The disease assignment of spending for outpatient services and drugs covered by MHI was based on claims data from SWICA, a major supplier of MHI with a market share of 8.1% in 2017. The SWICA insured population was fairly representative of the total insured population: the sample had a similar age-sex structure as the full population and average per capita spending in MHI was only slightly below the Swiss average. Moreover, two morbidity indicators showed that the sample was comparable to the general population. The proportion of the population hospitalized at least once as well as the proportion of the population with a nursing home stay by age groups and sex were very similar as the ones in the general population. A table in the supplementary material (Online resource 1) compares the two populations. Due to the lack of diagnostic coding in outpatient care, diseases were identified based on diagnostic clues included in claims data. These clues included disease-specific drugs or treatments and the specialization of the treating physician. Spending was assigned to diseases using direct assignment and regression-based methods. The methodology is described in Stucki et al. [14].

Spending for the treatment of *injuries* was based on the claims data from SWICA and accident claims provided by Suva, the largest supplier of accident insurance with a market share of about 50%. MHI insurers like SWICA provide accident insurance to those who do not have compulsory accident insurance under the Accident Insurance Act through their employer. The data allowed for a separation of injury-related spending from illness-related spending by MHI at the individual level. A further distinction by types of injuries was not possible.

The Suva claims hold information on the health service type, the type of damage (accident or occupational disease), the type of accident (at work/occupational or leisure time), a flag for traffic accidents, as well as sex and age of patients. We used pooled claims data from 2011 to 2013 for 2012 and from 2016 to 2018 for 2017 to assign spending to diseases. Total spending on each claim was summed up by condition and the resulting spending shares for each health service and by sex and age were applied to the total spending covered by the accident insurance as reported in the NHA.

Health care spending for the treatment of *congenital birth defects* up to age 20 is covered by mandatory disability insurance IV. NHA report the spending by IV for each health service. We directly assigned this spending to *congenital birth defects* in the age groups below 20 and further split by sex and age groups based on IV expenditure data provided by the Federal Social Insurance Office. Treatment of *congenital birth defects* above the age of 20 years is covered by MHI. It was not possible to identify the relevant outpatient treatments in MHI claims data due to a lack of specific diagnostic clues. We thus estimated spending on outpatient treatments of *congenital birth defects* above the age of 20 by assuming the same ratio between inpatient and outpatient care of those below 20 and those above 20 years. These spending estimates by service were subtracted from the MHI spending totals to avoid double counting.

Total dental care according to NHA, which was mainly financed out-of-pocket (78.9% in 2017) and by supplementary insurance (14.3%), was assigned to *oral disorders*. The distribution of out-of-pocket spending over sex and age groups was based on the frequency of dentist visits according to the SHS for those above age 15. Spending covered by supplementary insurance was assigned according to information by SWICA about the distribution of dental spending by sex and age of their population enrolled in supplementary insurance. Out-of-pocked financed dental care for those below the age of 15 was imputed from the SWICA data.

#### Inpatient acute somatic care

Disease identification was straightforward for inpatient somatic care, as HospReg includes ICD-10-GM coding of the main diagnosis and up to 49 secondary diagnoses for every inpatient stay. Except for treatments covered by accident insurance (which was assigned in the same way as the outpatient care spending) we assigned spending^1^ based on the case-specific DRG and the corresponding cost weights in HospReg. Unlike a previous study for Switzerland [15], we accounted for comorbidities, coded as secondary diagnoses. This procedure was developed in previous research [26]. As the actual impact of comorbidities on costs cannot be determined if only the DRG cost weight is known, we used similar case-level data from the cantonal health department of Zurich that also included the reported production costs for each case. HospRegZH is a sub-sample of the national HospReg holding this additional cost information. We estimated regression models for all cases with the same main diagnosis with the cost per case as the dependent variable and the up to 46 disease indicators for the comorbidities as the independent variables. We then reattributed part of the case costs from the main diagnosis to the comorbidities. Based on the model coefficients from the regression models in HospRegZH, we computed inflows (the cost part flowing in from that disease coded as comorbidity) and outflows (the cost part flowing out to comorbidities) for each disease in HospReg. Finally, we applied these inflows and outflows to the DRG cost weights in HospReg. The cost weight proportions attributed to each disease were multiplied with the service-specific spending according to NHA to obtain spending estimates.

#### Inpatient rehabilitation and psychiatry

Disease identification in inpatient rehabilitation and psychiatry was also based on ICD-10 codes in HospReg. We computed spending for each disease for both services based on the sum of the length of stay of all episodes where the disease was coded as the main diagnosis. By dividing this sum by the total sum of the length of stay of all episodes in rehabilitation and psychiatry, we obtained the spending share for each disease.

#### Inpatient long-term care

Disease identification in patients living in nursing homes was especially challenging, as there is no nationwide data collection of diagnoses affecting those patients. HospReg contains information about where a patient was admitted from and referred to after the inpatient stay. We used this information and combined it with the SWICA claims data. We selected all patients from HospReg in 2012 and 2017 who were not discharged to another hospital and defined a binary indicator variable equal to 1 if the patient was discharged to a nursing home for the first time (i.e., not admitted from a nursing home). This indicator was used as the dependent variable in a logistic regression model with disease indicators defined based on the main and the first secondary diagnoses as independent variables. We assessed the effect of each disease on the probability of being discharged to a nursing home and ranked the diseases according to the size of the regression coefficients. For each individual in the SWICA claims data who had positive nursing home spending, we determined from all clues-based diagnoses the main diagnosis based on the disease ranking from HospReg. Individuals who had the disease on rank 1 received that disease as main diagnosis. Individuals who did not have this disease received as main diagnosis the disease with the highest rank among all the diseases that were present in that individual. We assigned all the spending for nursing homes at the individual level to the main diagnosis.

### Methods of decomposition of disease-specific spending increases

We used the Das Gupta decomposition method for aggregate measures [27] to identify which factors accounted for the change in spending by diseases and other perspectives over time. The method corrects for compositional effects when comparing multiple populations, such as those from different years. We decomposed the observed spending difference between 2012 and 2017 into four additive components: population size, sex and age population structure, disease prevalence, and spending per prevalent patient.

The yearly aggregate spending can be written as the sum over the spending observed in each of the 42 age/sex groups (a) and 48 disease (d) cells. These costs are a product of the four factors:

$$spending=\sum_{d=1}^{48}\sum_{a=1}^{42}population*\frac{{population}_{a}}{population}*\frac{{prevalent}_{a,d}}{{population}_{a}}*\frac{{spending}_{a,d}}{{prevalent}_{a,d}}$$

where ${spending}_{a,d}$ is the sum of the spending over all 20 health services $\sum_{s=1}^{20}{spending}_{s}$.

We used prevalence rates by disease, age group, sex, and year from the GBD study [23] and combined it with population data from FSO [24]. For *injuries* and *neoplasms*, we used incidence instead of prevalence rates. Those were retrieved from insurers (*injuries*) and from the GBD (*neoplasms*). As *osteoporosis* was not included in the GBD, we used prevalence rates from the SHS [22]. Prevalence rates of ‘other’ (residual) disease categories and *well care* were taken from health insurance claims data (clues-based approach).

## Results

### Spending by disease in 2012 and 2017

We present the spending by disease for major disease groups (GBD level 2) and for specific diseases (GBD level 3).

The major disease groups with the highest spending in both years were *mental disorders* (2012: 16.1%, 2017: 14.3%), followed by *musculoskeletal disorders* (12.8%, 13.8%) and *neurological disorders* (9.4%, 8.5%). Table 3 shows the spending in million Swiss Francs (m CHF) as well as the spending shares in both years, along with the absolute and percentage increase in spending between 2012 and 2017.

**Table 3:** Spending by major disease groups (GBD level 2), percentage change 2012–2017 and spending shares by year

**Disease group (GBD level 2)**	**Spending in m CHF**		**Change 2012–2017**		**Spending shares (%)**	
	**2012**	**2017**	**m CHF**	**%**	**2012**	**2017**
Mental disorders	10,734	11,371	636	5.9	16.1	14.3
Musculoskeletal disorders	8497	11,007	2511	29.5	12.8	13.8
Neurological disorders	6240	6753	512	8.2	9.4	8.5
Injuries	5883	6681	798	13.6	8.8	8.4
Cardiovascular diseases	5240	6094	855	16.3	7.9	7.7
Other NCDs	4718	5303	585	12.4	7.1	6.7
Neoplasms	3685	5157	1472	39.9	5.5	6.5
Oral disorders	4414	4772	358	8.1	6.6	6.0
Communicable diseases	3005	3838	834	27.7	4.5	4.8
Sense organ diseases	2508	3607	1099	43.8	3.8	4.5
Digestive diseases	2710	3546	836	30.8	4.1	4.4
Well care	2278	2837	559	24.5	3.4	3.6
Prevention	1700	1937	237	14.0	2.6	2.4
Skin and subcutaneous diseases	1279	1717	438	34.2	1.9	2.2
Diabetes and kidney diseases	1120	1659	540	48.2	1.7	2.1
Nutritional deficiencies	790	1311	522	66.0	1.2	1.6
Chronic respiratory diseases	1047	1205	158	15.1	1.6	1.5
Maternal and neonatal disorders	665	847	182	27.4	1.0	1.1
Total health care spending	66,513	79,642	13,129	19.7	100.0	100.0

The highest increase between 2012 and 2017 was observed for *nutritional deficiencies* (66.0%). This increase was mostly driven by an increase in acute somatic inpatient spending and a rise in the number of prescriptions of iron supplementation in the outpatient setting. The spending growth was much below average for *mental disorders* (5.9%), *oral disorders* (8.1%), and *neurological disorders* (8.2%).

**Fig. 2:**
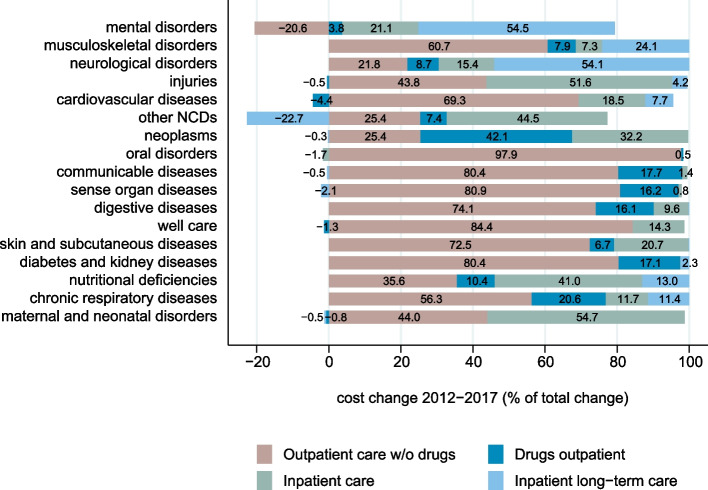
Spending increase for each major disease group (GBD level 2) by type of health service category

Figure 2 shows the percentage contributions of each of four aggregated health service categories to the total spending increase by disease. For many disease groups, the largest increase was observed in outpatient care. The only exception was *mental disorders*, which showed a decrease in outpatient care (including psychotherapy) over the period. Drugs in outpatient care contributed to the increase in spending in all disease groups except *cardiovascular diseases*, *well care,* and *maternal and neonatal disorders*.

Table 4 lists the single diseases at GBD level 3 and their spending share by year as well as the percentage change in spending between 2012 and 2017. Among the conditions with the highest spending share was *depression* (2012: 4.7%, 2017: 4.1%) and the residual *other* conditions within each level 2 disease category (e.g., *other communicable diseases*, 2012: 3.9%, 2017: 4.1%).

**Table 4:** Spending by disease, 2012 and 2017, percentage change 2012–2017 and share of aggregated health care services of spending by disease in 2017

**Disease (GBD level 2/3)**	**Spending in m CHF**		**Change (%)**	**Spending shares (%)**		**Service share (%) of total disease spending in 2017**				
	**2012**	**2017**	**2012–2017**	**2012**	**2017**	**outpatient**	**outpatient drugs**	**inpatient acute care**	**inpatient long-term care**	**other**
**Communicable diseases**										
HIV/AIDS	355	384	8.2	0.5	0.5	18.7	77.5	0.9	0.7	2.2
Hepatitis	24	164	589.0	0.0	0.2	8.0	87.7	1.8	0.8	1.8
Other communicable diseases	2626	3291	25.3	3.9	4.1	71.2	8.7	15.9	0.4	3.9
**Maternal and neonatal disorders**	665	847	27.4	1.0	1.1	32.4	0.1	65.3	0.5	1.6
**Nutritional deficiencies**	790	1311	66.0	1.2	1.6	33.5	12.4	25.4	25.9	2.7
**Neoplasms**										
Colon and rectum cancers	346	549	58.6	0.5	0.7	29.2	15.8	52.2	0.7	2.1
Trachea, bronchus, and lung cancers	397	734	84.6	0.6	0.9	17.6	49.9	29.3	1.2	2.0
Breast cancer	586	742	26.5	0.9	0.9	38.8	28.9	28.1	2.0	2.2
Prostate cancer	275	458	66.4	0.4	0.6	30.9	33.3	33.7	0.1	2.0
Other neoplasms	2081	2676	28.6	3.1	3.4	18.9	16.2	62.6	0.6	1.8
**Cardiovascular diseases**										
Ischemic heart disease	1100	1322	20.2	1.7	1.7	37.5	10.2	49.4	0.2	2.8
Stroke	872	1277	46.5	1.3	1.6	8.8	1.3	79.1	7.8	3.0
Hypertensive heart disease	52	51	-1.3	0.1	0.1	27.6	45.7	23.5	0.2	3.0
Atrial fibrillation and flutter	509	532	4.7	0.8	0.7	20.7	10.1	67.5	0.1	1.6
Other cardiovascular and circulatory diseases	2708	2912	7.5	4.1	3.7	45.9	25.9	25.0	0.2	3.0
**Chronic respiratory diseases**										
COPD	300	398	32.7	0.5	0.5	21.6	19.1	51.1	5.0	3.1
Asthma	418	408	-2.4	0.6	0.5	43.8	44.0	9.3	0.2	2.7
Other chronic respiratory diseases	329	399	21.1	0.5	0.5	28.3	13.4	55.8	0.1	2.4
**Digestive diseases**										
Cirrhosis and other chronic liver diseases	99	92	-7.4	0.1	0.1	13.4	1.2	81.0	2.2	2.2
Other digestive diseases	2611	3454	32.3	3.9	4.3	46.6	16.8	34.0	0.0	2.5
**Neurological disorders**										
Alzheimer’s disease and other dementias	1543	2126	37.8	2.3	2.7	6.1	3.8	6.2	82.3	1.6
Parkinson’s disease	1428	1243	-12.9	2.1	1.6	8.1	8.1	5.8	76.2	1.7
Epilepsy	1163	1196	2.8	1.7	1.5	33.1	15.8	11.5	36.0	3.5
Multiple sclerosis	200	317	58.7	0.3	0.4	13.7	62.7	11.1	9.2	3.3
Other neurological disorders	1907	1870	-1.9	2.9	2.3	52.3	12.6	12.3	18.0	4.8
**Mental disorders**										
Schizophrenia	600	568	-5.2	0.9	0.7	5.5	6.0	60.7	23.6	4.2
Depression	3145	3293	4.7	4.7	4.1	18.7	8.7	21.4	48.1	3.1
ADHD	200	219	9.3	0.3	0.3	52.0	33.6	7.9	1.3	5.2
Alcohol and drug use disorders	977	1067	9.1	1.5	1.3	3.6	1.5	42.4	36.1	16.5
Other mental disorders	5812	6224	7.1	8.7	7.8	27.2	6.7	13.9	48.8	3.4
**Diabetes and kidney diseases**										
Diabetes mellitus	682	1014	48.6	1.0	1.3	44.0	37.9	12.6	2.0	3.4
Chronic kidney disease	437	646	47.6	0.7	0.8	75.0	8.1	10.7	0.0	6.2
**Skin and subcutaneous diseases**	1279	1717	34.2	1.9	2.2	60.2	15.2	21.2	0.4	3.0
**Sense organ diseases**	2508	3607	43.8	3.8	4.5	72.6	14.1	5.0	4.7	3.7
**Musculoskeletal disorders**										
Rheumatoid arthritis	437	569	30.2	0.7	0.7	21.2	70.5	0.7	4.7	2.9
Osteoarthritis	1233	1459	18.4	1.9	1.8	20.2	5.7	70.1	0.9	3.1
Low back pain	282	420	48.9	0.4	0.5	61.7	4.9	26.2	2.6	4.6
Osteoporosis	2317	2963	27.8	3.5	3.7	18.4	10.7	2.5	66.3	2.2
Other musculoskeletal disorders	4227	5596	32.4	6.4	7.0	59.0	9.2	23.3	3.1	5.5
**Oral disorders**	4414	4772	8.1	6.6	6.0	92.8	0.5	0.5	0.0	6.2
**Other NCDs**										
Congenital birth defects	1851	1943	5.0	2.8	2.4	40.6	4.2	25.8	12.3	17.2
Other non-communicable diseases	2867	3359	17.2	4.3	4.2	27.9	16.1	53.7	0.1	2.2
**Injuries**										
Road injuries - occupational	40	57	42.1	0.1	0.1	35.6	1.4	53.2	0.1	9.7
Road injuries – non-occupational	370	443	19.7	0.6	0.6	44.6	1.7	44.0	1.1	8.6
Other injuries – non-occupational	544	612	12.4	0.8	0.8	54.3	3.0	33.9	0.5	8.2
Other injuries –occupational	834	1016	21.8	1.3	1.3	52.9	2.4	36.4	0.6	7.8
Residual injuries	4095	4553	11.2	6.2	5.7	16.7	0.4	46.2	32.9	3.9
**Well care**	2278	2837	24.5	3.4	3.6	71.7	0.2	25.1	0.0	3.0
**Prevention**	1700	1937	14.0	2.6	2.4	0.0	0.0	0.0	0.0	100.0

Only few diseases showed a decrease in spending over time. Examples are *schizophrenia* (-5.2%) and *Parkinson’s disease* (-12.9%). Spending for most diseases increased between 2012 and 2017. The largest increase was observed for *hepatitis* (+ 589.0%), which was driven by the approval of a new drug within that period. *Trachea, bronchus and lung cancer* also showed a strong rise in spending of 84.6%. *Prostate cancer* (+ 66.4%), *colon and rectum cancers* (+ 58.6%), and *multiple sclerosis* (+ 58.7%) also belonged to the conditions with increases of more than 50%.

### Spending by age, sex, and service

**Fig. 3:**
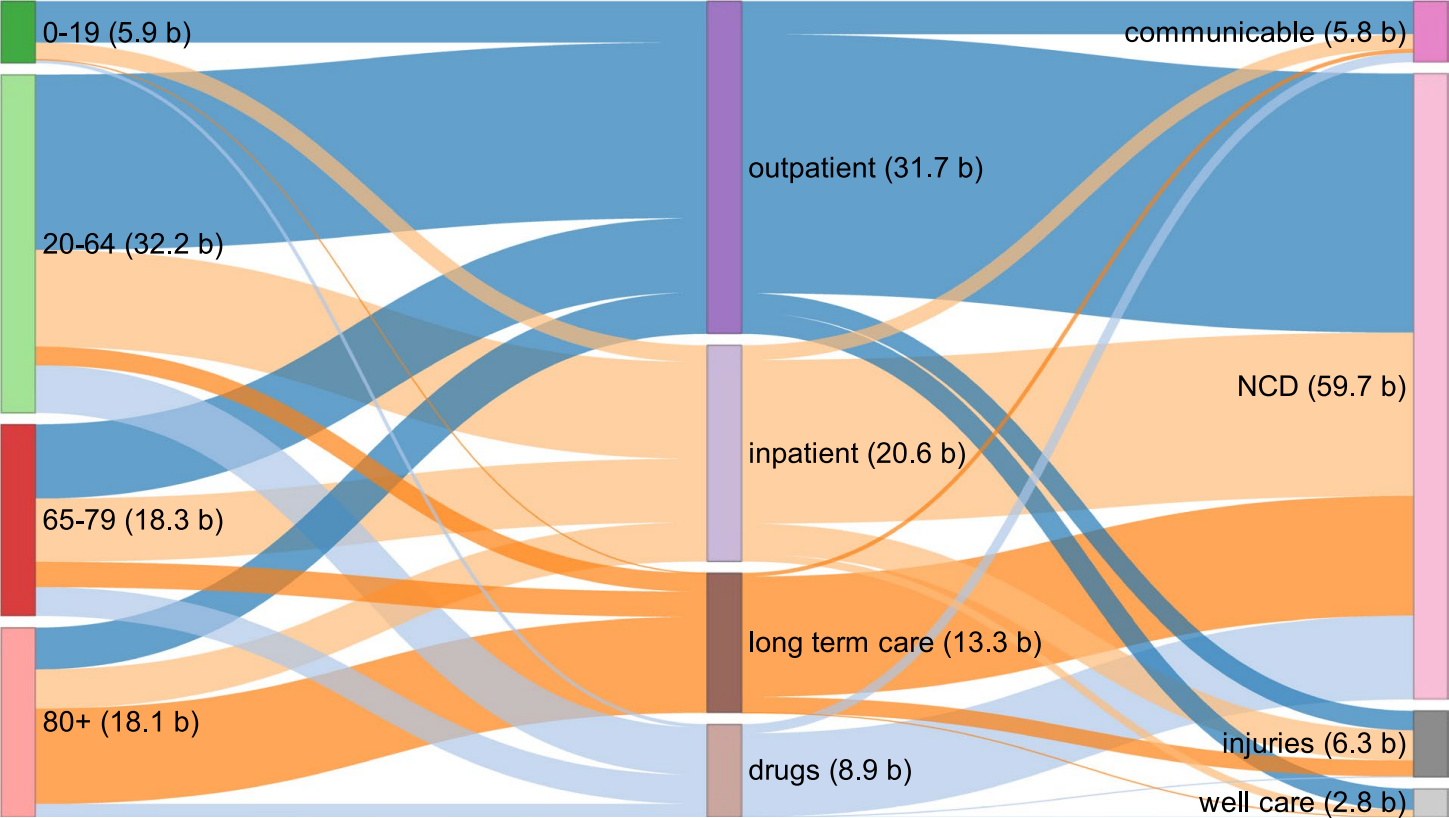
Spending in 2017 by age, health service, and disease (GBD level 1). b: billion; communicable diseases include maternal/neonatal disorders and nutritional deficiencies

Figure 3 shows the decomposition of total spending in 2017 by broad age categories, health service categories, and disease categories. The share of outpatient care of total spending was higher in younger individuals. The share of inpatient care, on the other hand, increased with increasing age. From a disease perspective, NCDs were the dominant category, accounting for 80.0% of total spending (excluding prevention).

**Fig. 4:**
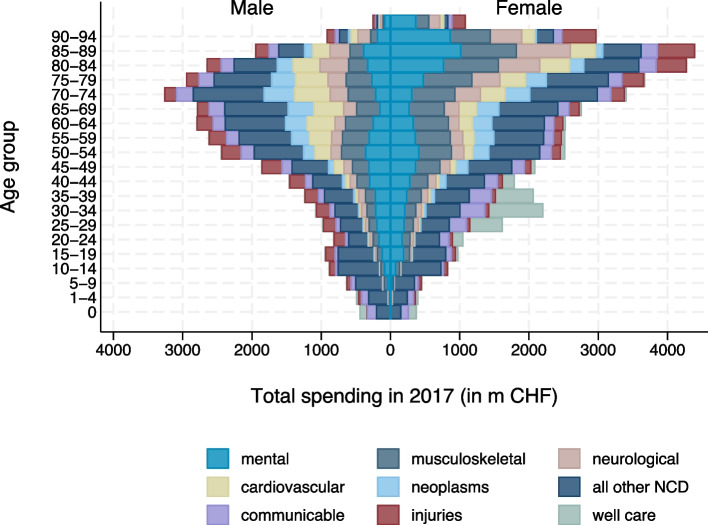
Spending by age, sex, and diseases (top 5 GBD level 2 NCD and the remaining GBD level 1 diseases)

The relevance of disease groups for total spending differed across age groups. Figure 4 displays the total spending by age group for the five NCD with the highest spending in 2017, along with the other NCD summed up in *all other non-communicable diseases* and the other three aggregated categories *injuries*, *communicable diseases* (including *maternal/neonatal disorders* and *nutritional deficiencies*), and non-diseases (*well care*). Total spending was highest in the age group 70–74 (men) and 85–89 (women), respectively. The higher spending in women in older age groups was mostly due to *neurological disorders* (such as *Alzheimer’s disease and other dementia*), as well as *mental disorders* and *injuries* (e.g., falls). *Well care* spending, which is mostly pregnancy-related spending, explains the differences in the spending pattern in the age groups up to age 40. Men in age groups below 65 had higher spending for *injuries* than women.

### Decomposition of the spending change between 2012 and 2017

We observed significant heterogeneity in the magnitude of spending changes over time across diseases (see Table 4). The decomposition of the changes in disease-specific spending showed that almost half (43.5%) of the aggregate spending increase between 2012 and 2017 was due to increases in spending per prevalent patient. The change in population size (29.8%), the change in population structure (14.5%) and the changes in the prevalence of the included diseases and injuries (12.2%) accounted for the remainder.

**Fig. 5:**
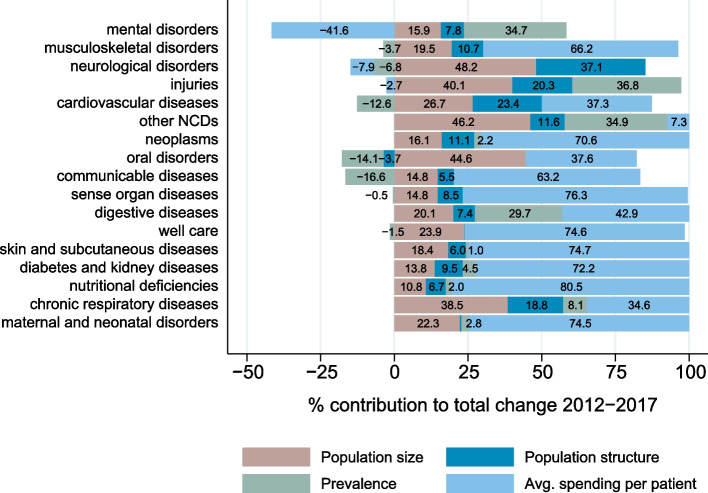
Factor decomposition results for major disease groups (GBD level 2; sorted by total spending share in 2017)

The relevance of each factor varied substantially between the disease groups (Fig. 5). For all but three disease groups (*mental disorders, neurological disorders, injuries*), spending per prevalent patient was associated with an increase in disease-specific spending. In most cases, it was also higher than the overall 43.5%. For *nutritional deficiencies*, the factor’s association with the total disease-specific spending increase was 80.5%. For *mental disorders* (-41.6% of total change), *neurological disorders* (-7.9%), and *injuries* (-2.7%), we observed a decrease in spending per prevalent patient.

The changes in prevalence were generally associated with spending increases in most cases, with the biggest contributions in *injuries* (36.8% of the total spending change), *mental disorders* (34.7%) and *digestive diseases* (29.7%). The association was negative for *communicable* (-16.6% of total change), *neurological* (-6.8%), *cardiovascular* (-12.6%), and *musculoskeletal diseases* (-3.7%), as well as for *well care* (-1.5%).

The association of the changing age/sex structure of the population with the disease-specific spending was highest for *neurological disorders* (37.1% of the total spending change) and *cardiovascular diseases* (23.4%). It was lower for diseases which were more frequently prevalent in younger age groups, such as *mental disorders* (7.8%) or *communicable diseases* (5.5%).

Table 5 lists the corresponding results for the single diseases at the GBD level 3. In neoplasms, spending per prevalent patient was the factor that was associated most with the spending increase over time. The associations ranged from 9.0% of spending in 2012 (*other neoplasms*) to 87.9% (*trachea, bronchus, and lung cancers*). Spending per prevalent patient was associated with a spending decrease for *hypertensive heart disease* (-17.2%) and *atrial fibrillation and flutter* (-10.8%), even though for their aggregate category *cardiovascular diseases* the association was positive. The contrary was true for *Alzheimer’s and other dementias* (+ 23.8%) and *multiple sclerosis* (+ 53.0%) within *neurological disorders*, for which the factor had a negative association with the spending change.

**Table 5:** Spending by disease in 2012 and spending change 2012–2017, in total and contributions by factors (in CHF and %-points of spending change)

**Disease (GBD Level 2/3)**	**Spending 2012**	**Total change**		**Population size**		**Population structure**		**Prevalence**		**Spending per prevalent patient**	
	m CHF	m CHF	%	m CHF	% points	m CHF	% points	m CHF	% points	m CHF	% points
***All conditions***	***64,813***	***12,872***	***19.8***	***3837***	***5.9***	***1869***	***2.9***	***1573***	***2.4***	***5593***	***8.6***
**Communicable diseases**											
HIV/AIDS	355	29	8.2	20	5.6	-1	-0.4	9	2.4	2	0.5
Hepatitis	24	140	589.0	5	20.8	1	3.8	0	0.2	134	564.2
Other communicable diseases	2626	665	25.3	159	6.1	69	2.6	-215	-8.2	652	24.8
**Maternal and neonatal disorders**	665	182	27.4	41	6.1	1	0.1	5	0.8	136	20.4
**Nutritional deficiencies**	790	522	66.0	56	7.1	35	4.4	10	1.3	420	53.2
**Neoplasms**											
Colon and rectum cancers	346	203	58.6	24	6.9	20	5.7	-24	-6.8	183	52.8
Trachea, bronchus, and lung cancers	397	336	84.6	30	7.7	24	6.2	-68	-17.1	349	87.9
Breast cancer	586	155	26.5	36	6.1	11	1.8	-60	-10.2	169	28.8
Prostate cancer	275	183	66.4	20	7.1	26	9.5	-15	-5.4	152	55.3
Other neoplasms	2081	595	28.6	128	6.1	82	3.9	199	9.6	187	9.0
**Cardiovascular diseases**											
Ischemic heart disease	1100	222	20.2	65	5.9	57	5.2	-5	-0.5	105	9.6
Stroke	872	405	46.5	58	6.6	58	6.7	-66	-7.6	355	40.8
Hypertensive heart disease	52	-1	-1.3	3	5.4	2	3.9	3	6.6	-9	-17.2
Atrial fibrillation and flutter	509	24	4.7	28	5.5	28	5.6	22	4.4	-55	-10.8
Other cardiovascular and circulatory diseases	2708	204	7.5	151	5.6	122	4.5	-98	-3.6	30	1.1
**Chronic respiratory diseases**											
COPD	300	98	32.7	19	6.3	19	6.3	-23	-7.6	83	27.8
Asthma	418	-10	-2.4	22	5.3	6	1.5	-11	-2.6	-28	-6.6
Other chronic respiratory diseases	329	70	21.1	20	6.0	4	1.3	47	14.2	-1	-0.3
**Digestive diseases**											
Cirrhosis and other chronic liver diseases	99	-7	-7.4	5	5.2	3	2.9	2	1.6	-17	-17.0
Other digestive diseases	2611	843	32.3	163	6.2	59	2.2	246	9.4	375	14.4
**Neurological disorders**											
Alzheimer’s disease and other dementias	1543	583	37.8	98	6.4	108	7.0	9	0.6	368	23.8
Parkinson’s disease	1428	-185	-12.9	72	5.1	85	6.0	5	0.4	-348	-24.3
Epilepsy	1163	32	2.8	64	5.5	37	3.2	14	1.2	-82	-7.0
Multiple sclerosis	200	117	58.7	14	6.9	-1	-0.5	-1	-0.7	106	53.0
Other neurological disorders	1907	-37	-1.9	102	5.3	40	2.1	-77	-4.0	-102	-5.3
**Mental disorders**											
Schizophrenia	600	-31	-5.2	32	5.3	4	0.7	2	0.3	-69	-11.5
Depression	3145	148	4.7	173	5.5	123	3.9	-30	-0.9	-118	-3.8
ADHD	200	19	9.3	11	5.6	-5	-2.7	1	0.6	12	5.8
Alcohol and drug use disorders	977	89	9.1	55	5.6	-6	-0.6	-24	-2.5	64	6.5
Other mental disorders	5812	412	7.1	328	5.6	181	3.1	1364	23.5	-1461	-25.1
**Diabetes and kidney diseases**											
Diabetes	682	331	48.6	45	6.6	30	4.3	27	3.9	230	33.7
Chronic kidney disease	437	208	47.6	29	6.6	22	5.0	-3	-0.6	160	36.6
**Skin and subcutaneous diseases**	1279	438	34.2	80	6.3	26	2.0	4	0.3	327	25.6
**Sense organ diseases**	2508	1099	43.8	164	6.5	94	3.7	-5	-0.2	847	33.8
**Musculoskeletal disorders**											
Rheumatoid arthritis	437	132	30.2	27	6.2	9	2.0	-8	-1.9	105	23.9
Osteoarthritis	1233	227	18.4	72	5.9	54	4.4	6	0.5	95	7.7
Low back pain	282	138	48.9	19	6.7	7	2.6	-9	-3.3	121	42.9
Osteoporosis	2317	624	26.9	142	6.1	135	5.8	-107	-4.6	455	19.6
Other musculoskeletal disorders	4227	1369	32.4	264	6.2	82	1.9	19	0.5	1003	23.7
**Oral disorders**	4414	358	8.1	247	5.6	-20	-0.5	-78	-1.8	209	4.7
**Other NCD**											
Congenital birth defects	1851	93	5.0	102	5.5	-30	-1.6	41	2.2	-20	-1.1
Other non-communicable diseases	2867	492	17.2	168	5.9	98	3.4	163	5.7	63	2.2
**Injuries**											
Road injuries - occupational	40	17	42.1	3	6.8	-1	-3.5	-8	-19.4	23	58.2
Road injuries – non-occupational	370	73	19.7	22	6.0	-6	-1.5	-41	-11.1	98	26.4
Other injuries – occupational	544	68	12.4	31	5.7	-2	-0.4	-31	-5.8	70	12.9
Other injuries – non-occupational	834	182	21.8	50	6.0	-6	-0.7	47	5.6	92	11.0
Residual injuries	4095	458	11.2	233	5.7	187	4.6	344	8.4	-306	-7.5
**Well care**	2278	559	24.5	137	6.0	0	0.0	-9	-0.4	430	18.9

*m* million

## Discussion

### Interpretation of disease-specific spending

This study estimated disease-specific health care spending by age, sex, and health services in Switzerland in 2012 and 2017. We found that *mental diseases* accounted for the highest share of spending, followed by *musculoskeletal disorders* and *neurological disorders*.

We estimated the direct medical spending for treatment of a disease which may differ from the overall medical spending triggered by a disease. The case of *diabetes* illustrates this point, as diabetes is a well-known risk factor for *cardiovascular diseases*, *sense organ diseases* and other diseases. From an etiological perspective the spending triggered by diabetes is likely to be higher than our estimate of 1.3% in 2017.

Tracking disease-specific spending over time helps to understand the drivers of spending. While the total spending increased by 19.7% between 2012 and 2017, the growth rates at the disease level were heterogenous. Interestingly, *cardiovascular diseases* showed a decrease in total outpatient drug spending over time. One possible explanation is a decrease in drug prices due to patent expiration, leading to a decrease in spending, even without a change in the number of treated patients.

For certain conditions, the increase in drug spending was a major driver of disease-specific spending. That was especially true for diseases for which new drugs were introduced within the 5-years period, such as grazoprevir for *hepatitis* or nivolumab for *lung cancer*.

Spending on *Alzheimer’s and other dementias* is likely to increase further with ageing population [28]. The same is true for other diseases like *stroke* and *hypertensive heart disease*, for which age is an important risk factor. We were able to show that the changing age/sex structure was associated with a spending increase of 6–7% in *stroke*, *Alzheimer’s and other dementias* and *Parkinson’s disease*.

Our results also show which services are most affected by the demographic transition and the associated changes in the disease burden. One example is *Alzheimer’s and other dementias,* for which spending arises mostly in inpatient long-term care (share of 82.3% of spending in 2017). Accordingly, most of the spending increase was due to that type of care. In contrast, spending for *cardiovascular diseases* arises mostly in somatic inpatient care (e.g., *stroke* or *atrial fibrillation and flutter*) and drugs (e.g., *hypertensive heart disease*).

### Comparison with previous studies

Our general cost-of-illness approach differs from the many single cost-of-illness studies which focus on only one disease. There are some studies for Switzerland and other developed countries which took a similar approach.

Table 6 compares the results of the present study with a study for the US by Dieleman et al. [11] and a previous study for Switzerland by Wieser et al. [15]. Both studies used the GBD disease classification. The US study decomposed spending from 1996 to 2016 using the full GBD level 3 disease classification [11, 12] while the Swiss study decomposed spending in 2011 by major diseases at GBD level 2. We limit the comparison to diseases included in all studies and exclude spending for prevention (Switzerland) and the treatment of risk factors (US). The spending shares for the present study thus differ slightly from those reported in the Results section of this paper.

**Table 6:**
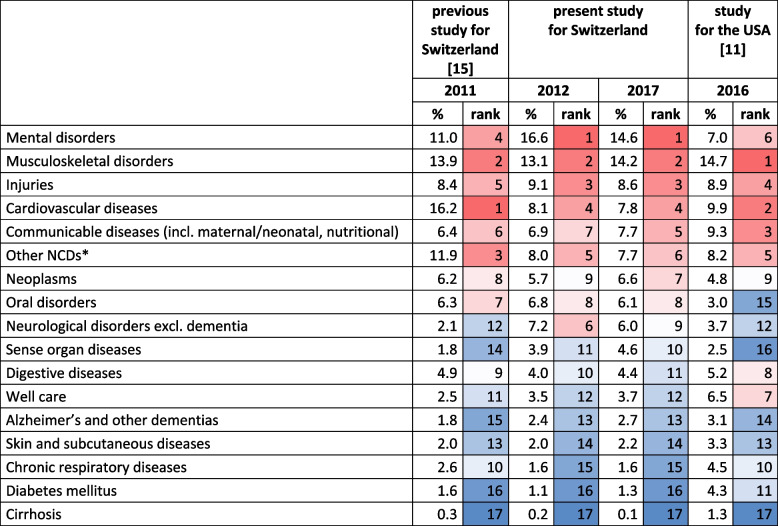
Comparison with the results of similar studies

Swiss studies without prevention, US study without treatment of risk factors

Diseases are ordered according to their rank in the present study in 2017

^*^Other NCDs include urogenital, blood, endocrine disorders, and congenital defects

We compare the results of the US study with our results for 2017, as it is closest to 2016. The six top conditions were the same in both countries, but the ranking differed. The US study assigned 14.7% of total spending to *musculoskeletal disorders* (14.2% in our study). We assigned a lower share (7.8%) to *cardiovascular diseases* than the US (9.9%), which might be driven by differences in prevalence rates. In comparison to the US, we found a higher spending share of *neoplasms* (6.6% vs. 4.8%), *sense organ diseases* (4.6% vs. 2.5%), and *oral disorders* (6.1% vs. 3.0%). On the other hand, our spending estimates were lower for *chronic respiratory diseases* (1.6% vs. 4.5% in the US) and *well care* (3.7% vs. 6.5%), which may be due to a broader definition of *well care* in the US study. We also found a lower spending share of *diabetes* (1.3% vs. 4.3%). Different factors such as racial and ethnical composition, socio-economic disparities, and health behavior [29] might contribute to this difference.

*Mental disorders* were the top condition in Switzerland (spending share of 14.6%) but accounted for only 7.0% of US spending. This important difference might be related to more limited access to mental health care in the US. Recent results from Norway are more similar to our spending estimates. Kinge et al. (2023) found that mental disorders accounted for 20.7% of health care spending in Norway in 2019 [30]. Roehrig (2016) concluded that mental disorders were the costliest group of conditions in the US in 2013 [31]. However, it is important to note that this study included dementia in the mental disorders category, which makes a comparison of results difficult. A substantial part of spending for *mental disorders* in Switzerland occurred in inpatient long-term care (see Table 4). Consequently, the total spending depended heavily on that service type. As the attribution of spending for long-term care to diseases was based on sparse data, there is some uncertainty around the spending estimate for *mental disorders*.

The previous Swiss study had a similar scope and decomposed total NHA health care spending in 2011 by 21 major diseases. We compare it to our results for 2012, as it is closest to 2011. The most striking difference is the substantially higher spending for *cardiovascular diseases*, with spending share of 16.2% vs. 8.1% in the present study. This difference is probably driven by methodological differences in the spending assignment in outpatient care, where the previous Swiss study focussed on high-prevalence conditions and thus tended to overestimate the spending for these. The remaining spending estimates were surprisingly similar, e.g., *injuries* (8.4% vs. 9.1% in the present study), *neoplasms* (6.2% vs. 5.7%), or *skin and subcutaneous diseases* (2.0% vs. 2.0%).

There are other studies with a similar scope but a different decomposition framework. Rachas et al. (2022) decomposed French health care expenditures in the years 2015–2019 by 58 diseases [32]. For 2019, the French study estimated a similar spending share for *mental disorders* (14.0% vs. 14.3% in our study for 2017), and higher spending shares for *cardiovascular diseases* (14.0% vs. 7.7%), *chronic respiratory diseases* (2.1% vs. 1.5%), and *neoplasms* (12.0% vs. 6.5%). However, the comparison with the study is problematic as it assigned 22% of spending to ‘hospitalizations for other reasons’ and 6.7% to ‘no condition’. Moreover, the study did not report spending for *musculoskeletal diseases*. These limitations highlight the importance of an exhaustive decomposition framework. Interestingly, the study found a very similar yearly growth rate for *lung cancer* (11.4%/year vs. 13.0%/year in our study), the disease with the highest relative spending increase in our study except for hepatitis.

### Interpretation of spending drivers

The results of the decomposition of spending increases from 2012 to 2017 by four spending drivers must be interpreted against the background of constant prices of health care services and products over the entire study period. According to FSO price statistics the overall index of health care prices even fell by 2.5% over the 5-year period [33]. The spending increases attributed to the four spending drivers can thus be interpreted as increases in the amount of health services consumed.

The overall spending increase of 19.7% over the 5-year period corresponds to an annual growth rate of 3.7%, which can be split into the annual contributions of the four spending drivers. Spending per prevalent patient was the most important driver with an annual growth rate of 1.6% or a share of 43.5% in overall spending increase. Population growth was the second most important driver with a growth rate of 1.1%. Subtracting this from the total growth rate we obtain a yearly per capita growth rate of 2.6%. Changes in the population sex-age composition and changes in disease prevalence contributed 0.5 percentage points each to the yearly growth rate.

The interpretation of spending per prevalent patient is less straightforward than the interpretation of population growth, population structure, and disease prevalence. This factor is also more relevant for policy makers, as the other three drivers can hardly be influenced by health policy.

The following factors may be influencing the spending per prevalent patient: *First*, patients may be receiving newly developed procedures and drugs, which are more expensive than the standard of treatment. *Second*, the intensity of treatment with existing procedures and drugs may be increasing (intensive margin). In both situations, it is crucial for health policy to evaluate if the increases in spending per patient were cost-effective, i.e., led to better health outcomes at reasonable costs. Comparing the change in spending per patient and the change in the disease burden is an interesting extension of the disease-specific spending estimation that has recently been studied by several researchers in the United States [9, 34]. In cases in which spending increases do not lead to much additional value for patients, it may be appropriate to take measures against the volume expansion.

### Contributions

Our study addressed several shortcomings of the previous research and contributed to the literature in three ways. *First*, by decomposing overall health spending at a more granular level it generated a detailed description of spending for many important diseases. *Second*, by including the perspectives of sex and age it generated important insights into the distribution of disease burden across demographic groups. *Third*, and most importantly, by evaluating the spending decomposition across 2 years it allowed for the disaggregation of the spending increase by four underlying cost drivers.

Spending decompositions by disease may be particularly useful for the evaluation of the health care system performance when complemented by health outcome data on productivity growth at the disease level [9] or by spending-effectiveness ratios (e.g., spending per disability-adjusted life year averted) [34]. Such analyses can provide answers to whether health care spending growth for specific diseases pays off in terms of health gains or not.

### Limitations

This study has several limitations. *First*, the lack of diagnostic coding in ambulatory care impedes the identification of many diseases in health insurance claims data. In contrast to many comparable studies [11, 12, 30], we did not have access to diagnostic information at the level of single encounters. Our use of diagnostic clues in claims did not allow for the identification of diseases with unspecific treatments, such as *low back pain* or *osteoarthritis*. Our study is thus likely to substantially underestimate the spending on these specific diseases. For diseases with similar treatments, we might mis-allocate spending (e.g., *asthma* and *COPD*, which are hard to distinguish based on medication only). Moreover, our approach leads to an overestimation of spending for the residual *other* conditions within each GBD level 2 category. As an example, *other mental disorders* accounted for more than half of spending for *mental disorders* in 2017. While we were confident about attributing spending to GBD level 2 categories, a further attribution to specific diseases (e.g., *depression*) was not always possible. Consequently, *other mental disorders* may contain both spending for “real other” conditions (e.g., anxiety) and spending for the four specific conditions within *mental disorders*.

A *second* limitation is related to changes in the diagnostic clues in the claims data over time. The introduction of new disease-specific drugs and treatments may increase the number of patients identified with a disease, even if the overall number of patients has not changed. In our study this might have been the case with *hepatitis C* or *lung cancer*, which saw the introduction of new drugs between 2012 and 2017. This effect may have contributed to the strong increase of spending per prevalent case.

A *third* limitation is related to the lack of data for other perspectives in the decomposition, such as the sex and age structure for certain service types. We did not have access to the spending distribution by sex and age for out-of-pocket payments. In most cases, we used the sex and age structure from other health care services for which we had high-quality micro-data. For others, we derived the sex/age structure from other data (e.g., the SHS).

A *fourth* limitation is due to missing micro-data for the assignment of spending for inpatient long-term care. Due to this lack of diagnostic coding, we based our spending estimation on a combination of claims data and inpatient registry data. However, the HospReg data only covers the part of the institutionalized population that was hospitalized. We assumed that diseases identified in these patients were equally likely to lead to nursing home admissions in the non-hospitalized patients. However, the link between the use of long-term care and specific diseases might be less straightforward than for other health services, as need of care may be caused by general frailty.

*Fifth*, the effect of comorbidities on spending may be more complex than we were able to capture with our methodology. We accounted for comorbidities whenever possible but were not able to include interaction terms in the regression-based assignment due to the type and amount of data at our disposal. However, treatment costs for a patient suffering from two diseases may deviate significantly from the sum of spendings of two patients with one of the diagnoses each. This is a potentially major limitation of the regression approach used in this study.

*Sixth*, not all data sources used in the estimation were necessarily representative of the full population. The potential lack of representativeness of the health insurance claims data is a major limitation of our study. It covered only around 10% of the population in both years. As we scaled up the disease-specific spending to the total given in the NHA, this could lead to a potential bias. A comparison of several indicators, including the payments into the risk equalization fund, the proportion of the population with hospitalization or nursing home stay, the age-sex structure, and per capita spending, in the sample and in the general population suggests that our data was fairly representative of the total population. However, the study sample may be less representative based on other, unobserved indicators of morbidity.

*Finally*, our decomposition of disease-specific spending over time did not include the number of individuals actually treated, but only the estimated overall prevalence according to GBD estimates for Switzerland. These estimates include both treated and untreated individuals. This limitation must be taken into account when interpreting the spending per prevalent patient.

### Future research

The change in the average spending per prevalent patient is driven by multiple factors, such as price changes, medical progress (i.e., new services), or intensity of treatment at the intensive margin (more of the same treatments for the same individuals) and extensive margin (extension of the treatment to previously untreated individuals). From a health policy perspective, a further decomposition of this factor would be highly relevant, as it would reveal the underlying drivers of spending growth.

Furthermore, future research should address the limitations mentioned above. One major improvement would be to include a measure of treated prevalence in the decomposition. This would enable the distinction of an epidemiological measure (overall prevalence) from a health service provision measure (share of prevalent patients treated). Another important improvement would be to include claims data from several health insurers. This would lead to a higher precision as the number of individuals used in the bottom-up estimation of spending would increase.

Finally, a spending decomposition at the sub-national level would be useful for health policy makers and provide a tool to explain differences in health care spending across regions.

## Conclusions

At present, little is known about how much single diseases contribute to total health care spending in Switzerland and on the relative importance of potential drivers of spending growth. We decomposed total health care spending by a comprehensive and mutually exclusive set of diseases and services in 2012 and 2017. Our results show that *mental*, *musculoskeletal*, and *neurological diseases* accounted for more than one third of total health care spending in both years. The reasons for the change in disease-specific spending over time varied significantly across diseases. Notably, we observed a decrease of the average spending per prevalent patient for *mental* and *neurological diseases*. For most other diseases, the rising spending per patient led to an increase in disease-specific spending.

Spending decompositions by diseases and other perspectives may be particularly important from a health policy perspective, as they may indicate areas for cost containment policies. Moreover, decomposing the spending change over time into the contribution of underlying factors can guide the definition of global spending budgets currently discussed in Switzerland and elsewhere, as well as health care provision planning. Finally, disease-specific spending estimates at a granular level and at different points in time can serve as an input to system-wide cost-effectiveness studies, which would be useful for value-based health policy.

### Supplementary Information

**Additional file 1.** Details about methodology.

## Data Availability

The data that support the findings of this study are available from the institutions named in the study, but restrictions apply to the availability of these data, which were used under license for the current study, and so are not publicly available. Request for access to the data from the Swiss Health Survey can be sent to sgb@bfs.admin.ch. Request for access to the inpatient registry data can be sent to gesundheit_dsv@bfs.admin.ch. Request for access to claims data from SWICA Health Care Organisation CH can be sent to maria.trottmann@swica.ch. Request for access to claims data from Suva accident insurance can be sent to unfallstatistik@suva.ch. Request for access to the other data sources can be sent to the corresponding author (stcc@zhaw.ch). Access to data from SWICA Health Care Organisation CH (claims data), the Federal Statistical Office (inpatient registry and Swiss Health Survey), the cantonal health department of Zurich (inpatient registry), Suva accident insurance (claims data), and the disability insurance/Federal Social Insurance Office (claims data) requires a data use agreement with the data provider.

## Abbreviations

ADHD: Attention Deficit Hyperactivity Disorder
b: Billion
CHF: Swiss Franc
CHOP: Swiss classification of surgical interventions
COPD: Chronic obstructive pulmonary disease
DRG: Diagnosis-related group
FSO: (Swiss) Federal Statistical Office
GBD: Global Burden of Disease
GDP: Gross Domestic Product
GM: German modification
GP: General practitioner
HospReg: Hospital inpatient care registry
HospRegZH: Inpatient hospital registry from canton of Zurich
ICD: International Classification of Diseases
IV: Disability insurance
LTC: Long-term care
m: Million
MHI: Mandatory health insurance
NCD: Non-communicable diseases
NHA: National Health Accounts
SHS: Swiss Health Survey
STATPOP: Swiss population statistics
US: United States

## Footnotes

1 The spending includes cantonal subsidies to hospitals for teaching and research (“Gemeinwirtschaftliche Leistungen” GWL). Based on NHA data, we split these subsidies across inpatient acute somatic care, inpatient rehabilitation, and inpatient psychiatry. GWL were assigned to diseases top-down using the spending shares resulting from the bottom-up estimation.
