# Supplementary material for: What drives health care spending in Switzerland? Findings from a decomposition by disease, health service, sex, and age
Source: BMC Health Serv Res. 2023 Oct 25;23:1149. doi: 10.1186/s12913-023-10124-3 (PMC10598929; doi:10.1186/s12913-023-10124-3)
Supplement: Supplementary file 1 — Additional file 1. Details about methodology. [file 12913_2023_10124_MOESM1_ESM.pdf]

Additional file 1: Details about methodology

Article title: What drives health care spending in Switzerland? Findings from a decomposition by disease, health service, sex, and age

Authors: Michael Stucki, Xavier Schärer, Maria Trottmann, Stefan Scholz-Odermatt, Simon Wieser

Journal: BMC Health Services Research

Corresponding author:

Michael Stucki

ZHAW Zurich University of Applied Sciences

Winterthur Institute of Health Economics

Gertrudstrasse 8

8401 Winterthur

Switzerland

Email: stcc@zhaw.ch

Phone: +41 58 934 40 76

ORCID: 0000-0002-2595-1238

Total spending by service

Table 1 shows the total health care spending by services and year according to the National Health Accounts by the Federal Statistical Office (FSO).

Table 1 Total health care spending by health services and year

		Spending according to NHA (m CHF)	
**Service category**	**Service**	**2012**	**2017**
Outpatient	Physician services (general practitioners GP)	2,754	3,860
	Physician services (specialist)	3,795	4,489
	Hospital outpatient	4,717	6,307
	Drugs outpatient	7,328	8,906
	Psychotherapy and psychiatry	923	1,301
	Physiotherapy	1,015	1,636
	Occupational therapy	123	194
	Dental care	4,171	4,473
	Medical devices and products	2,333	2,671
	Long-term home-care	1,882	2,566
	Other outpatient care	565	679
Other outpatient care	Laboratory tests	1,131	1,871
	Radiology	843	1,132
	Ambulance and rescue	346	460
Inpatient care	Acute somatic care	12,926	14,313
	Rehabilitation	1,695	1,834
	Psychiatry	1,771	1,917
	Long-term care in nursing homes	11,950	13,376
Administration	Administration	2,899	3,157
Prevention	Prevention	1,700	1,937
**Total**		**66,512**	**79,643**

Source: National Health Accounts (FSO)

Spending assignment

Outpatient care spending covered by MHI

The methodology used to assign spending in outpatient care to diseases follows the study by Stucki et al. (2021) [1]. It was applied to the following health care service types:

- general practitioners (GP)
- specialist physicians
- outpatient hospital
- drugs (prescribed and over the counter)
- home care (outpatient long-term care)
- physiotherapy
- occupational therapy
- outpatient psychiatry
- laboratory tests performed by external laboratories outside the doctor's office
- radiology
- dental care (for the few indications covered by mandatory health insurance)
- and other spending (e.g., devices)

The study distinguished between 42 diseases, which is the same as in the current study, excluding the five *injury* conditions as well as *prevention*.

Data sources

We used individual-level claims data for 709,788 individuals in 2017 and 558,703 individuals in 2012. The data contains detailed information about spending in mandatory health insurance by health service. Spending includes co-payments and deductibles. The data also contain selected single billing positions which were used in the identification of diseases.

The samples cover about 10% of the insured population in both years. Data are roughly representative of the Swiss general insured population, as a comparison of some indicators shows (see Table 2).

Table 2 Comparison of study sample from SWICA and the general population

	**Study sample**	**General population**
Proportion of population hospitalized		
Female, 0-19 years	3.8%	3.9%
Female, 20-44 years	10.4%	10.8%
Female, 45-64 years	8.1%	8.3%
Female, 65+ years	19.6%	20.7%
Male, 0-19 years	4.0%	4.3%
Male, 20-44 years	3.3%	3.5%
Male, 45-64 years	8.0%	8.4%
Male, 65+ years	21.4%	22.6%
Proportion of population in inpatient long-term care		
Female, 65-69 years	0.8%	0.7%
Female, 70-74 years	1.6%	1.5%
Female, 75-79 years	4.1%	3.4%
Female 80-84 years	10.1%	8.7%
Female, 85-89 years	20.7%	20.1%
Female, 90+ years	36.5%	41.5%
Male, 65-69 years	0.5%	0.7%
Male, 70-74 years	1.3%	1.2%
Male, 75-79 years	2.7%	2.4%
Male 80-84 years	6.1%	5.3%
Male, 85-89 years	11.6%	11.4%
Male, 90+ years	22.0%	25.6%
Age/sex distribution (% of total population)		
Female, 0-19 years	9.4%	9.7%
Female, 20-44 years	18.0%	16.5%
Female, 45-64 years	15.0%	14.0%
Female, 65+ years	8.7%	10.1%
Male, 0-19 years	10.0%	10.3%
Male, 20-44 years	17.0%	17.0%
Male, 45-64 years	14.5%	14.2%
Male, 65+ years	7.5%	8.1%
Spending (per capita, Swiss Francs)		
Total gross MHI spending (unweighted)	3461	3849
Total gross MHI spending (weighted)	3551	3849
Outpatient gross MHI spending (unweighted)	2602	2834
Outpatient gross MHI spending (weighted)	2652	2834

Note: Data from 2017. The proportion of population hospitalized in the general population was computed from the inpatient hospital registry (containing all inpatient episodes in Swiss hospitals) [2] and population statistics by the Federal Statistical Office (Population and Households Statistics (STATPOP)) [3]. The proportion of population in inpatient long-term care in the general population was taken from Obsan [4]. Numbers from Obsan only refer to the population in nursing homes at the end of each year. We were not able to determine the nursing home status at the end of the year in the SWICA data and therefore excluded all patients who died in the year from the sample before computing the proportions of the population in nursing

homes. The weights used in the computation of the mean spending per capita were used to adjust for differences in the age/sex structure. The mean spending in mandatory health insurance (MHI) was taken from the Statistics on the Compulsory Health Insurance 2017 (Statistik der obligatorischen Krankenversicherung 2017) by the Federal Office of Public Health [5].

Disease identification based on 'diagnostic clues'

Diseases were identified based on 'clues' in the claims data, namely specific billing positions from the following national tariff catalogues:

- The TARMED (tarif médical) for physician services (either technical (e.g., thorax MRI) or time-dependent services (e.g., 5 minutes of consultation))
- The AL (Analysenliste) for laboratory tests
- The MiGel (Mittel- und Gegenständeliste) for therapeutic devices such as hearing aids
- The SL (Spezialitätenliste) for drugs; classification according to the hierarchical anatomical therapeutic chemical (ATC) classification of the World Health Organization.
- The SwissDRG catalogue for inpatient acute care

The DRG does not necessarily correspond to only one disease according to our disease classification. We checked the degree of correspondence between the diseases from our list and the DRG codes in the inpatient registry HospReg (Medizinische Statistik der Krankenhäuser) [2]. Whenever a DRG code was 95% specific to one disease, we used it as a single clue in the identification.

The disease identification algorithms consist of single clues (e.g., specific billing positions), or a combination of clues (e.g., a specific billing position and physician specialization). When clues allowed for a disease identification only at GBD level 2 but not at level 3, individuals were assigned to the residual 'other' disease category at level 3.

Direct spending assignment

In each year, we observed spending for each individual i by service s, $y_{i,s}^{total}$.

The spending decomposition by disease involved two steps: a direct and an indirect (regression-based) assignment.

Spending for clues that were used in the disease identification was assigned directly to that disease, e.g., specific drugs for diabetes. This resulted in $y_{i,s,d}^{direct}$ for each individual and disease.

Regression-based spending assignment

The residual spending $y_{i,s}^{residual} = y_{i,s}^{total} - \sum_{j=1}^{42} y_{i,s,j}^{direct}$ at the individual level was assigned to diseases based on regression models.

We ran regressions of spending on all 42 disease indicators, separately for 56 groups that were defined based on seven outpatient service types, sex (male/female), and age (4 groups: <20 y./20-44 y./45-64 y./65+ y.). Three service types (physiotherapy, psychiatry, dental care) were fully attributed to a disease group. For two services (home care and occupational therapy), we did not use the regression-based approach to assign spending in the second step.

We estimated a Poisson pseudo-maximum likelihood (PPML) model and used the coefficients for the estimation of attributable fractions (AF) and spending shares for each disease:

$$AF_{i,s} = \frac{\widehat{y_{i,s}} - e^{\alpha_s}}{\widehat{y_{i,s}}}$$

$$s_{i,s,d} = \frac{\left(e^{\beta_{s,d}} - 1\right) * I_{i,d}}{\sum_{j=1}^{42}\left[\left(e^{\beta_{s,j}} - 1\right) * I_{i,j}\right]}$$

The part of the residual spending at the individual level assigned to any disease d was obtained by multiplying the AF with the spending share and the residual spending:

$$spending_{i,s,d} = AF_{i,s} * s_{i,s,d} * y_{i,s}^{residual}$$

Total national spending for each combination of disease, service, sex, and age group was obtained by multiplying the spending shares from the claims data analysis with the total spending by MHI for each service according to NHA. We weighted the spending using sex and age specific weights to account for the slightly different structure of the SWICA and the general Swiss population in both years.

Outpatient and inpatient care spending covered by accident insurance scheme

Data sources

Data on healthcare spending were retrieved from the administrative Suva database. This database is fed directly from the electronic billing systems of the service provider. The database comprises a table with header information of the invoices (e.g., the service provider or total amount invoiced) and a linked table with details on the items on the invoice (e.g., tariffs catalogue, the specific tariff item, and the amount invoiced for this service). For invoices which were not received via the electronic billing system, data from the Suva healthcare cost statistical database were used. This register database is structured in a similar way as the administrative database, but with data originating from manual data entry. Accident-related data (e.g., age category, sex, or occupational/non-occupational accident) are taken from administrative data [6]. The diagnoses, type of injury, and the flag for road traffic accidents are taken from a sample for which cases have been labeled manually as described in more detail in [7]. Costs have then been extrapolated from this sample.

The sample covers Suva accidents with accident registration dates back to 1984, older cases had to be excluded. Only negligible costs of older accidents will be excluded by doing so.

Analysis dimensions

The costs were aggregated along the following dimensions for the analysis:

- The time ranges of arrival of the invoices from 2011-2013 and 2016-2018
- Sex of patient
- Age of patient at the time of the accident, 5-years age categories
- Type of injury (according to GBD) as derived from the cases' diagnoses. ICD-10 main diagnose codes were used to determine the GBD category for each case. Injuries were classified at GBD level 3, while the other diseases were only classified at GBD level 2. This was done to account for the much smaller number of cases with diseases in our base set.
- Service type was derived from the tariff types (e.g., physiotherapy)
- Service provider. For the hospital and rehabilitation clinics, an additional distinction between inpatient and outpatient treatments was made based on a combination of the type of service provider and the tariff that was applied in the invoice.
- Type of damage (accident or occupational disease).
- Type of accidents (occupational [=BUV] or non-occupational (leisure time) accident [=NBUV] or accident insurance for unemployed [=UVAL])
- Flag for traffic accidents

Aggregation

The sum of the healthcare spending was calculated for the above dimensions, with extrapolation from the sample to the total. All data were joined and aggregated in one step, so that no individual data were extracted from the data sources at any time.

Combined information on diagnoses and claims data were only available for a sample of all accidents. While this randomized sampling process should not lead to systematic errors, extrapolation from a sample always introduces a random statistical sampling error into the analysis. Considering the numerous dimensions of analysis, this statistical error may be considerable when analyzing smaller cells or subgroups. We mitigated the effect of these unwanted statistical errors by pooling claims data over three-year time windows centered around the year of interest (i.e., 2011-2013 for 2012 and 2016-2018 for 2017).

There was a 3% discrepancy between the total health care costs and the extrapolation of health care costs for which invoice data were available. This difference is partly due to older cases, partly due to the extrapolation, but mostly due to accounting procedures. Transfers of costs between cases, transfers to other insurances, reversals and cancellations are not trackable with respect to which service provider and health services from our available database and were ignored in our analysis.

A correction was made to allow for invoices with missing data on the line-item level: When the sum of all line items in a subsegment did not add up to the total amount of the invoices for this subsegment, then the difference was distributed amongst the health services proportionally.

Accident insurers cover mostly spending due to injuries, but not exclusively. We included claims with other diseases but aggregated them at GBD level 2 and redistributed them to GBD level 3 conditions after upscaling the spending to the total paid by accident insurers.

Acute somatic inpatient care spending covered by all payers except for accident insurance

We applied a methodology previously used by Dieleman et al. (2017) for comorbidity adjustment in inpatient cases in the United States [8] and adapted it for our study. The goal of the comorbidity adjustment was to correct the case-level costs in acute somatic care by the presence of comorbidities. We distributed total health care spending for acute inpatient services, including the part covered by the supplementary private hospital insurance, based on this method.

Data sources

We used data from HospReg, which contains all inpatient episodes in every Swiss hospital for 2012 and 2017 (dataset 1). The data contains one main diagnosis (MD) and up to 49 secondary diagnoses (SD) according to the international classification of diseases (ICD-10) for each case, as well as age and sex of the patient and some information that allows for the classification of each case into acute somatic, rehabilitation, or psychiatric care. For acute somatic care, the data also contains the DRG cost weight. We kept acute somatic cases for which the DRG weight was non-missing.

In addition, we used a very similar data set containing a sub-sample of the cases that were treated in hospitals in the canton of Zurich (the largest region in Switzerland with about 1.5 m inhabitants) (HospRegZH; dataset 2). This second data set contains production costs for each case, i.e., the costs that arose in the treatment (for medical treatment, pharmaceuticals, care, accommodation etc.).

We mapped the ICD-10 codes of all acute somatic care cases in both data sets to our GBD disease classification to obtain the aggregate disease group of the MD as well as 46-1=45 disease indicators for the presence of a disease as a SD.

We used dataset 2 for the estimation of the coefficients from regression models as described in the next paragraph, and dataset 1 for the computation of the probabilities p_{kj} of two diseases being coded in the same case. The advantage of using case-level production costs instead of the DRG cost weights (remuneration) was that the variation of costs across cases was maintained.

Regression models and attributable fractions

For each main diagnosis k, we estimated regression models with the logarithm of the total case costs as the dependent variable and the binary disease indicator variables for the comorbidities j as the dependent variables:

$$ln(case\ costs_k) = \beta_{0k} + \sum_{j=1}^{J} \beta_{kj} comorbidity_{kj} + \varepsilon_k$$

If $\beta_{kj} > 0$, the comorbidity increases costs of a case with k as MD, if $\beta_{kj} < 0$, the comorbidity decreases costs of a case with k as MD. The model thus estimates the relative risk of higher spending due to the comorbidities.

We estimated separate regression models for four age categories: 0-14 / 15-44 / 45-64 / 65+ years.

Using these coefficients, we modelled the attributable fraction (AF) for each disease. The AF is the part of observed case costs that is due to each comorbidity. It is the product of the relative risk and the probability p_{kj} of the MD k and the comorbidity j being coded together in the same case:

$$AF_{kj} = p_{kj}(e^{\beta_{kj}} - 1)$$

Comorbidity restrictions

Some conditions were not allowed as comorbidities in cases with certain MD or SD. In the cases falling under the restrictions, we deleted the diagnosis before aggregating the codes into our set of diseases. This means that we applied the restrictions to the specific ICD-10 codes or groups of ICD-10 codes. We mostly followed the restrictions set by Dieleman et al. (2017), who used the full GBD Level 3 disease classification containing about 200 disease codes.

The restrictions in Table 3 were applied.

Table 3 Restrictions applied in the comorbidity adjustment in inpatient care

**Main diagnosis**	**Restrictions**
Any	The following diseases are not allowed as comorbidities:<br>• No endocrine, metabolic, blood, and immune disorders<br>• No "other" residual conditions (from the original GBD level 3 disease classification)
Any except lower and upper respiratory infections	No lower and upper respiratory infections as comorbidities
All neoplasms	No comorbidities allowed
Any	No *well care* as comorbidity
Any	No injuries as comorbidities

Inflows and outflows

For each disease, we computed inflows, i.e., the extra resources caused by this disease as a comorbidity in cases with another MD, and outflows, i.e., the resources taken away from cases in

which it was coded as the MD, but comorbidities were present and had an effect on the resource use. Resources per case in dataset 1 were captured by the DRG case cost weights.

Inflows were obtained as follows:

$$inflow_k = \sum_{j=1}^{J} (sum\ of\ case\ weights_j * AF_{kj})$$

Outflows were obtained as follows:

$$outflow_k = sum\ of\ case\ weights_k * \sum_{j=1}^{J} AF_{kj}$$

The sum of total inflows equals the sum of total outflows. For each disease, the netflow was defined as the difference between inflows and outflows:

$$netflow_k = inflow_k - outflow_k$$

Adjustment scalars and computation of spending shares

We added the netflow to the sum of DRG cost weights that would have resulted from a "MD-only" assignment in which the full DRG cost weight is assigned to the MD. We then divided the sum of DRG cost weights after adjustment by the sum of DRG cost weights before adjustment to obtain adjustment scalars. A value above 1.0 means that netflow was positive.

Finally, we summed up the cost weights after adjustment and computed each disease's spending share by dividing its sum of cost weights after adjustment by the sum of the cost weights across all diseases.

Inpatient rehabilitation and psychiatry

Disease identification in inpatient rehabilitation and psychiatry was based on ICD-10 codes in HospReg. As coding quality is considered substantially lower in rehabilitation, we replaced the main diagnosis in cases which followed an acute somatic inpatient stay within the three weeks prior to the rehabilitation clinic stay with the main diagnosis from the acute somatic treatment. Inpatient rehabilitation and psychiatric care are reimbursed based on national payment schemes that define severity-adjusted rates for each day. However, the information on the actual spending for each case was not available.

Inpatient long-term care spending

We combined information from two data sources to estimate spending for inpatient long-term care in nursing homes by disease.

Data sources

HospReg contains two variables indicating where a patient was admitted from and where the patient was discharged to after the inpatient stay. We kept all patients who were not discharged to another hospital and created for each patient a binary indicator variable equal to 1 if the patient was not admitted from a nursing home but referred to a nursing home after the inpatient stay, and equal to 0 for all other patients. We kept the ICD-10 codes of the main diagnosis and the first secondary diagnosis of every patient in the sample and mapped it to our disease classification.

The second data source was the SWICA claims data. It contains total spending for inpatient long-term care at the individual level. Furthermore, it contains the diseases identified based on the 'diagnostic clues' approach described above and in [1].

Regression-based ranking of diagnoses

Using the HospReg data, we estimated each disease's impact on the probability of being referred to a nursing home after the inpatient stay. We estimated three logit models for each year to allow for different effects in three broad age categories (<65 years / 65-74 years / 75+ years). The newly created indicator variable described above was defined as the dependent variable. The disease indicator variables were used as independent variables.

Few diseases were not allowed as independent variables, as we assumed that they would not causally affect the probability of a nursing home admission. These were: all *communicable diseases*, *maternal and neonatal disorders*, *skin and subcutaneous diseases*, *oral disorders*, and *well care*.

The coefficients from the regressions were used to rank the diseases according to their effect on being admitted to a nursing home, i.e., the disease with the largest coefficient was ranked 1, and the disease with the smallest coefficient was ranked last. Only diseases with positive coefficient which were statistically significant at the 5% level were kept for the ranking.

Table 4 shows the ranking of diseases for each of the three age categories and by year (sorted by the ranking in age group 75+ in 2017).

Table 4 Ranking of diseases based on logistic regression modelling of nursing home admission probability

	**2012**			**2017**		
**Disease**	**<65 y.**	**65-74 y.**	**75+ y.**	**<65 y.**	**65-74 y.**	**75+ y.**
Alzheimer's and other dementia	23	1	1	21	1	1
Osteoporosis	24	4	4	23	6	2
Other injuries	18	18	3	19	13	3
Parkinson's disease	22	5	2	22	3	4
Other mental disorders	6	7	5	6	7	5
Nutritional deficiencies	12	12	6	12	9	6
Epilepsy	8	6	10	11	5	7
Stroke	5	9	7	9	12	8
Depression	14	13	8	14		9
Other neurological disorders	10	10	9	10	10	10
Multiple sclerosis	2	3	16	5	4	15
ADHD	3	21	14	3	19	16
Schizophrenia	1	2	15	1	2	17
Cirrhosis and other chronic liver diseases	7	11		4	11	
Alcohol and drug use disorders	4	8		2	8	
Colon and rectum cancers				17		
Chronic kidney disease	13	15		16		
Diabetes mellitus	17			13		
COPD	11	16		7	14	
Other non-communicable diseases			12			
Other neoplasms	16			15		
Trachea, bronchus, and lung cancers	9	17		8	15	
Congenital birth defects	15			18		

Determination of main diagnoses and spending assignment

Finally, we determined for each individual in the claims data with positive spending for inpatient long-term care the main diagnosis for that service. Individuals were assigned the disease as main diagnosis which was ranked highest in the regression approach.

Example: Individuals in age group 75+ years which were identified as having *Alzheimer's and other dementias* in 2017 were assigned this disease as their main diagnosis. Individuals in the same group without that condition, but with *osteoporosis*, were assigned this disease as their main diagnosis, etc.

We assigned the total spending for inpatient long-term care to the main diagnosis. By dividing each disease's assigned spending by the total spending for inpatient long-term care in the claims data, we obtained the overall spending shares. These shares were applied to the total for this service type according to the National Health Accounts to obtain spending by disease. Consequently, we also used the age/sex structure of inpatient long-term care spending from the claims data. In the scaling to the national level, we weighted the spending by the same age/sex-specific weights as in [1].

Prevention and administration

We had no micro-data allowing a bottom-up disease-specific spending assignment for the two remaining service types 'prevention' and 'administration' and thus applied a NHA based top-down approach. Payer-specific administrative spending was distributed proportionally to the disease-age-sex spending resulting from the bottom-up assignment. Prevention spending was assigned the disease condition *prevention* without further decomposition by age or sex. We excluded the prevention spending from subsequent analyses that required spending estimates by all perspectives (age, sex, and disease).

Note on precision of estimates based on samples of the population

The high number of cells (i.e., combinations of year, age group, sex, service type, and disease) means that the number of observations used to estimate spending in each cell can be low. This might in some cases lead to a lack of precision. The problem is less pronounced in cases in which we have complete data, such as spending covered by disability insurance as well as all spending for inpatient services. Conversely, the problem may be more pronounced where our data covers only a sample of the population, as is the case for accident insurance (sample of about 50% of all insured) and health insurance claims data (sample of about 10% of all insured).

Regarding accident insurance, about 2000 cells per year (5% of all cells) were filled using only the information from the accident claims data sample. The decomposition process involved algorithms for fitting the sum over different aggregation levels (e.g., the service type), which are known with high precision and accuracy. The spending in each cell was extrapolated to the total of health expenditures covered by Suva; it deviated less than 3% from the known total. As we used pooled data from three years for the distribution of spending in one year, we calculated the relative error from our estimates compared to the estimates using only one year of data; it was 3.6% on average. Relative errors were naturally larger for cells with a low number of observations and low spending than for larger cells with high spending. The resulting absolute errors are small; they cannot be fixed, but their effect is mitigated by the normalization to the marginal sums. Given that the sample covered a large proportion of the total population, we considered our estimates to be sufficiently precise.

Regarding MHI, a total of 21,168 potential cells per year (slightly more than 50% of all cells) were fully or partially filled using the insurance claims data from MHI.

- 29.7% of all cells were empty by definition, because some diseases cannot affect some individuals (e.g., prostate cancer in females) or because a certain service type is not relevant in the treatment of some diseases (e.g., psychiatric care in osteoporosis).
- 24.3% of all cells were empty because there were no observations.
- In the remaining 46.0% of cells, there were a median of 307 observations per cell, which we considered sufficiently high. In 83.3% of the non-empty cells based on MHI claims, the estimations were based on at least 30 observations. 91.9% of the cells contained more than 10 observations, and 28.8% contained more than 1000 observations.

References

1. Stucki M, Nemitz J, Trottmann M, Wieser S. Decomposition of outpatient health care spending by disease - a novel approach using insurance claims data. BMC Health Serv Res. 2021;21(1):1264. doi:10.1186/s12913-021-07262-x.
2. Federal Statistical Office. Inpatient Registry 2012 and 2017 (Medizinische Statistik der Krankenhäuser 2012 und 2017). 2020.
3. Federal Statistical Office. Population and Households Statistics (STATPOP). 2021.
4. Obsan (Swiss Health Observatory). Inanspruchnahmerate von Alters- und Pflegeheimen. 2023. https://ind.obsan.admin.ch/indicator/obsan/inanspruchnahmerate-von-alters-und-pflegeheimen. Accessed 13 September 2023.
5. Federal Office of Public Health. Statistics on the Compulsory Health Insurance (Statistik der obligatorischen Krankenversicherung). 2020.
6. Spinnler D, Scholz S. Kapitel "Heilkosten". In: Suva, editor. Unfallstatistik UVG 2003-2007. Luzern 2009.
7. Scholz-Odermatt SM, Luthi F, Wertli MM, Brunner F. Appendix to: Direct health care cost and work incapacity related to complex regional pain syndrome in Switzerland: a retrospective analysis from 2008 to 2015. Pain medicine. 2019;20(8):1559-69.
8. Dieleman JL, Baral R, Johnson E, Bulchis A, Birger M, Bui AL et al. Adjusting health spending for the presence of comorbidities: an application to United States national inpatient data. Health Economics Review. 2017;7(1):1-10.
